# Soil transmitted helminth infection in primary school children varies with ecozone in the Ngorongoro Conservation Area, Tanzania

**DOI:** 10.1186/s41182-021-00310-6

**Published:** 2021-03-10

**Authors:** Manar Eltantawy, Karin Orsel, Ashley Schroeder, Domenica Morona, Humphrey D. Mazigo, Susan Kutz, Jennifer Hatfield, Mange Manyama, Frank van der Meer

**Affiliations:** 1 Department. of Production Animal Health, Faculty of Veterinary Medicine, University of Calgary, Calgary, AB Canada; 2 Department of Community Health Sciences, Cumming School of Medicine, University of Calgary, Calgary, AB Canada; 3 Department of Ecosystem and Public Health, Faculty of Veterinary Medicine, University of Calgary, Calgary, AB Canada; 4 Department of Parasitology, Catholic University of Health and Allied Science, Mwanza, Tanzania; 5 Division of Medical Education, Weill Cornell Medicine - Qatar, Doha, Qatar

**Keywords:** Soil transmitted helminths, Stool, Elementary schools, Sanitation practices, Diagnostic techniques, Elevation, BMI, Ngorongoro Conservation Area, Tanzania, Strongyloides spp

## Abstract

**Background:**

Soil-transmitted helminthiasis is a neglected tropical disease, thriving in environments of poverty and disadvantage. Our objective was to determine the prevalence and intensity of four soil transmitted helminths (STH) among primary school children in the Ngorongoro Conservation Area (NCA), Tanzania.

**Methods:**

A cross-sectional study was performed between May 15^th^ and June 2^nd^, 2014. Six of 20 primary schools were chosen, based on elevation, designated low elevation ecozone (LEE) or high elevation ecozone (HEE). A total of 340 children from standards one to four were recruited. Height and weight of each child was determined and body mass index (BMI) was calculated. Stool samples were analyzed using the Kato-Katz, Wisconsin, and Baermann techniques to detect STH eggs and larvae. An interviewer-administered questionnaire on socio-demographic variables was used to capture information from the school’s headmaster and a checklist was used to assess sanitation facilities.

**Results:**

STHs identified included *Ascaris* (presumably *lumbricoides*), *Trichuris* (presumably *trichiura*), hookworms (presumably *Ancylostoma duodenale* and/or *Necator americanus*), and *Strongyloides* (presumably *stercoralis*). The overall prevalence of STH infection was 29.0% in LEE and 34.0% in HEE. Prevalence was 34.3% and 28.2% in males versus females, respectively. *Ascaris* sp*.* were only identified in HEE, prevalence of *Trichuris* sp. and hookworms were significantly higher in HEE compared to LEE, and *Strongyloides* spp. prevalence was lower in HEE. Intensity of *Trichuris* sp. was significantly higher in HEE and lower for hookworms. We did not detect a significant relationship between BMI and helminth intensity; however, BMI was lower in lower elevations and in males vs. females. Sanitation practices are taught at the schools, but challenges were identified when implementing. Latrine facilities were available and latrine-cleaning routines were practiced; however, hand washing practices were challenging due to restricted water availability.

**Conclusions:**

Significant differences in prevalence in HEE and LEE exist, and STH infections are still very common among school children suggesting that anthelminthic intervention and education may be necessary in this region. Based on this outcome, the study area in the NCA would be classified as a medium risk area, where periodic treatment recommendations should be based on prevalence estimations in the different ecozones.

**Trial registration:**

Ethics approval was obtained from the Catholic University of Health and Allied Sciences (CUHAS; Lake Zone Institutional Review Board MR/53/100/307)); the Conjoint Health Research Ethics Board (CHREB) at the University of Calgary in Canada (Study ID REB14-0127); the National Institute of Medical Research (NIMR) of Tanzania; and the Tanzania Commission for Science and Technology (COSTEC).

## Background

Soil-transmitted helminthiasis is recognized as a neglected tropical disease by the World Health Organization (WHO), thriving in environments of poverty and disadvantage [1]. An estimated 2 billion people are parasitized with soil-transmitted helminths (STHs) worldwide, the majority of whom reside in low- and middle-income countries (LMIC) [2]. Clinical signs of severe STH infections include, but are not limited to, abdominal pain, diarrhea, blood and protein loss, and rectal prolapse [3, 4]. Notably, an additional burden can be expected during subclinical infections leading to stunting, reduced body mass index (BMI), and impact on cognitive abilities [5].

Ingestion of infective parasite eggs from the environment are principle routes for transmitting roundworms (e.g., *Ascaris lumbricoides*) and whipworms (e.g., *Trichuris trichiura*). Skin penetration of infective larvae is the most common route of transmission for hookworms (e.g., *Ancylostoma duodenale* and *Necator americanus*) and threadworms (e.g., *Strongyloides stercoralis*). These four types of nematodes comprise the majority of STH infections; however, other parasites such as cestodes, trematodes, and protozoa may also be present and cause disease [4]. STH thrive in the warm and damp soils of tropical and subtropical countries, leading to high prevalence of STH in many African countries, especially in communities with challenged socio-economic settings [6, 7].

According to the WHO, more than 610 million children of school age are at risk of morbidity due to STH-infections; however, WHO only lists *A*. *lumbricoides*, *T*. *trichuria*, *N*. *americanus*, and *A*. *duodenale* as the core STH species, not including, for example, *S*. *stercoralis* and the other helminths [1]. Children are in a period of intense physical growth and rapid metabolism, resulting in high requirements for nutrients and measurable impacts if nutrition is impaired. A potential lack of awareness of personal hygiene and tendency to play in contaminated soil puts children at higher risk of exposure. Therefore, infections can result in growth and cognitive retardation, malnutrition, stunting, and anemia. School attendance rate and school performance (reduced attention) can also be negatively impacted [8].

In Africa, > 89 million children are estimated to be parasitized with STHs, with many infected with at least two STH species [7, 9, 10]. To allow the easiest approach to treatment of large numbers of school-aged children, schools are often targeted to participate in interventions either to prevent or treat STH parasitism.

Tanzania is classified in the group of so-called LMICs in Eastern Africa and climate, rural lifestyle, and socio-economic status all contribute to a high risk for school children to contract STH infections [11]. The ministry of health/education in Tanzania provides elementary schools with annual albendazole treatments for children; however, it is unknown if the implementation of this program is equally successful across the country [12]. Information concerning prevalence, intensity, and diversity of STHs infections is scarce for most of Tanzania, especially for the Ngorongoro Conservation Area (NCA). Most studies in Tanzania focus on infections with *Plasmodium* spp. and Schistosomiasis. In the nearby lake zone, prevalence based on Kato-Katz (KK) testing are 12.6% for hookworm, 3.2% *A*. *lumbricoides*, and 0.008% for *T*. *trichiura* and, in the Magu district, 0% for *A*. *lumbricoides* and only 0.2% for *T*. *trichiura* [13, 14]. Two other studies that focused on coastal regions of Tanzania report prevalence estimations based on KK of 1.1% for *T*. *trichuris*, 6.1% for hookworms, and 4.2% for *S*. *stercoralis* [15], and an overall 6.1% STH prevalence in the Muheza district after 10y of mass drug treatment implementation [16].

The NCA is a remote rural district of the Arusha region in Northern Tanzania, with 21 wards home to an estimated population of 90,000 inhabitants, mainly of the Maasai tribe. They keep their cattle, goats, and sheep in a pastoralist lifestyle, to ensure optimal utilization of land resources aimed at maximizing production [17]. Maasai communities live in a clanship polygamy, where most of the family members live very closely in one big household also called bomas [18].

Due to cultural traditions, e.g., using their hands in eating and walking barefoot, Maasai may be at higher risk of STH exposure [19], as risk is closely associated with contaminated water, poor sanitation, poor hygiene, and poverty [20]. The NCA district has a substantial altitudinal climatic gradient of 600 m at the caldera’s floor in the NCA crater, up to 2200 m at the crater rim. This results in different ecozones, with low elevation characterized by primarily scattered bushes and short grassy plains and dry hot environments, whereas forests are dominant at high elevations and the climate is cooler with more rain fall and humidity [21]. Environmental conditions can also influence parasite abundance and diversity, with cool moist environments generally being more conducive to parasite survival and transmission than dry hot environments, although some parasite species are better adapted to different extremes in temperatures and/or humidity [22]. The aim of this study was to describe and compare the prevalence, intensity, and polyparasitism of four common STH infections in primary school children in two ecozones of the NCA region.

## Materials and methods

### Study area

This study occurred in the NCA in Northern Tanzania. The area has two rainfall periods, short rains from September to December and long rains from March to May/June. The low elevation ecozone (LEE) is 1000 to 1900 m above sea level, with an annual rainfall of 500 to 700 mm and mean monthly temperatures of 17–19 °C. In contrast, the high elevation ecozone (HEE) is characterized by a cool, wet ecozone at > 1950 m above sea level with an annual rainfall of 800–1200 mm, and lower mean monthly temperatures of 12–14 °C [23].

### Study design and population selection

The district education officer (DEO), district medical officer (DMO), and NCA dispensary health workers were consulted and asked to prepare a list of all primary schools in the NCA for this study. Six primary schools were chosen and visited between May 15^th^ and June 2^nd^, 2014, based on two criteria. Firstly, no anthelmintic drug treatment was provided through school-based mass medication, to the students in the 6 months prior to the study. Secondly, schools in LEE had to be ~ 1000 to 1990 m above sea level, and HEE at ~ 2000 and 3000 m above sea level. Sample size estimations to determine the STH prevalence with imperfect tests [24] were combined with a cluster sampling strategy [25], as test objectives are clustered by ecozones. Using this information, the minimum number of schools enrolled was 6 primary schools in the NCA. The sample size for the number of children that were enrolled per school was determined using the methodology recommended by the WHO. This included working with a minimum threshold of 50 school children from each school, regardless of elevation or region [26, 27]. Accordingly, 300 children from standards one to four (ages 8–12 years, with a focus on ages 8–10 to limit age variation) were recruited based on parental consent and the children's willingness to participate.

We included six primary schools of which three schools were in the LEE, whereas the other three schools were situated in the HEE. Coordinates and elevations for each school were recorded with a hand-held GPS device. The research team visited each school to explain study objectives and procedures with the school headmaster and teachers and to distribute parental consent forms prior to sample collection. Teachers were tasked to explain the study to the parents. The consent form was written in Kiswahili and when necessary, teachers translated it into the local language (e.g., Maa). The sampling visit was scheduled 1 week later.

### Field procedures

Each school was visited by a team consisting of one local elder, a minimum of two local translators of both sexes, and members of the Tanzanian and Canadian university research team. Upon arrival at a school, team members provided communal instructions (in Kiswahili and Maa) regarding the objectives of the study and procedures to be followed. They also provided opportunities for children to ask questions. Thereafter, researchers assembled into three teams, with distinct responsibilities. At the registration station, the children’s school, sex, and age were recorded, the latter of which was reported by teachers. At the measurement station, children’s height and weight were measured twice and averaged. Stool sample containers were handed out to each child with a paper plate and wooden scoop for self-sample collection. Children were then directed to the latrine to deposit and collect a fresh sample. At the stool collection station (two sub-stations, one for females and one for males), children returned their stool sample to the team, and sanitized their hands. Samples were stored in bags before transportation.

### Laboratory procedures

Stool samples were brought to the Endulen Hospital laboratory the morning of collection. Samples were processed using modified Wisconsin [28] and beaker Baermann techniques [29]. To detect helminth eggs, the Wisconsin technique was modified as follows: feces (5 g) were homogenized in water with a tongue depressor. The stool solution was poured through a single layer cheesecloth mesh into a cup; and the filtrate transferred to a 15 mL test tube with Sheathers solution and centrifuged at 1500 rpm for 5 min with a glass coverslip on top of the test tube [20]. After centrifugation, the cover slip was lifted straight up and placed onto a microscope slide. Parasite eggs with a specific gravity less than that of the Sheathers solution floated to the top and were collected on the coverslip. This test has a high sensitivity for eggs of three species of STH: *Ascaris* spp., *Trichuris* spp., and hookworms [28]. A quantitative Baermann technique was used to detect *Strongyloides* spp. larvae. We adapted the Baermann method as described by Forrester et al. [29]. Briefly, 5 g of feces was weighed, placed in a cheesecloth envelope, and suspended below the water surface in a 250 mL beaker filled with tap water for at least 8 h (mostly overnight), enabling larvae to move into the water. The fecal packet and supernatant were removed, leaving approximately 15 mL of sediment which was collected in its entirety and stored in 96% ethanol (minimum 5:1 ratio) for subsequent microscopic examination at the laboratory of the Catholic University of Health and Allied Sciences (CUHAS) in Mwanza. Parasite eggs and larvae were identified based on morphology [30, 31]. Rhabditiform first stage larvae were identified in the Baermann sediment based on larval morphology and morphometry consistent with that described by Little, 1966 [32].

For quality-control purposes, ~ 10% of samples for males and females from each school were also processed using the Kato-Katz technique (Vestergaard Frandsen, Lausanne, Switzerland), considered by WHO as a reference test for detecting STH eggs. However, as this test has a reported low sensitivity, it was only included to report prevalence based on this reference test for comparative reasons [33–35]. All samples were processed according to the manufacturer’s instructions. A sample was considered positive for helminth infection if at least one helminth egg was present in the Kato-Katz smears or in the Wisconsin analyses, and/or if a larva was present in the Baermann assay. Presences and identification of eggs and/or larvae was determined by AS, who was trained and supported by parasitologists SK/DM and HM (all co-authors).

### Headmaster interviews

The research team conducted interviews on site with the school headmasters to collect information on the student and teacher population, the number of classrooms, water supply, existing food program, the deworming program, as well as sanitation and hygiene education (Appendices 1 and 2).

### Latrine inspections

Latrine facilities were evaluated using an observation checklist that included: types of latrines, cleanliness of the floor, the presence of fecal matter outside the facility, and presence of hand washing facilities (Appendix 2).

### Data analysis

Data were analyzed using the Statistical Package for Social Science (SPSS) software (Version 26.0). Descriptive statistics, such as frequencies, proportions, means, medians, and standard deviations, were computed. As described by [36], prevalence indicates the number of hosts from which at least one parasite egg/larva was isolated divided by the total number of examined hosts and expressed as a percentage; and intensity is the average eggs or larvae per gram of stool tested of a specified parasite in the hosts infected with this parasite [36]. Body mass index was calculated as weight in kilograms divided by the square of height in meters (kg/m^2^) and interpreted for the age and sex category [37, 38]. Chi-square (χ^2^) tests were used to detect statistical differences between categories of explanatory variables and replaced with Fisher’ exact tests if cell counts < 5. A regression model was used to explore associations between sex, ecozone, and BMI of children with helminth intensities, using ecozone as a fixed effect and exploring interactions between ecozone and sex.

## Results

### Study population

A total of 340 NCA-primary school children, from 6 schools were enrolled in the study. One sample was removed as the sex was not indicated on the form. Table 1 describes the participants demographics.

**Table 1:**
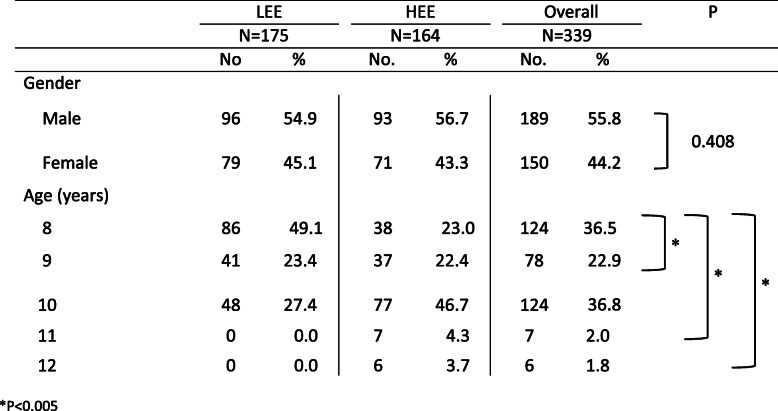
Demographic status of primary school children in low and high elevation ecozones in the NCA, Tanzania

Briefly, there was a significant difference between the number of children per age group enrolled between the LEE and HEE for age 8 vs. 9, 11 and 12 (*P* < 0.005).

The BMI of participants was within the WHO-identified normal range for age and sex in 89.4% of the male and 96% of the female participants, respectively. In a univariate comparison, a significant lower BMI was observed in the LEE, as well as in the males compared to females (*P* = 0.005 and *P* = 0.029 respectively; Table 2).

**Table 2:** BMI classification by sex presented per ecozone

	LEE^a^ *N* = 175	HEE *N* = 164			Overall *N* = 339	
	No	%	No	%	No	%
Male^b^	*N* = 96		*N* = 93		*N* = 189	
Severe thinness^c^	1	1	0	0	1	0.5
Thinness	9	9.4	10	10.8	19	10.1
Normal	86	89.6	83	89.2	169	89.4
Female	*N* = 79		*N* = 71		*N* = 150	
Severe thinness	0	0	0	0	0	0
Thinness	6	7.6	0	0	6	4.0
Normal	73	92.4	71	100	144	96.0

^a^Fisher exact *P* value between ecozones *P* = 0.005

^b^Fisher exact *P* value between sex *P* = 0.029

^c^Based on WHO definitions [37]

### Parasitological results: prevalence

Parasites were identified to the lowest taxonomic classification possible based on morphology. The STH detected included: *Ascaris* (presumably *lumbricoides*), *Trichuris* (presumably *trichiura*), hookworms (presumably *Ancylostoma duodenale* or *Necator americanus*), and *Strongyloides* (presumably *stercoralis*) (Table 3). Other parasites typically not considered STH were also documented and quantified and included eggs of a pinworm (*Enterobius* spp.; Prevalence 2.7% [0.7–4.7]) unspecified trematode (Prevalence 0.3% [0.3–1.1]) unspecified cestode (prevalence 6.0% [3.2-8.9]), and oocysts of *Entamoeba* spp. (prevalence 2.0% [0.3-3.8]) and *Isospora* spp. (Prevalence 0.3% [0–1.2]).

**Table 3:** Summary of prevalence (95% confidence interval) of four soil transmitted helminths with respect to ecozone and sex of children in the Ngorongoro Conservation Area

	*Ascaris* sp*.*	*Trichuris* sp.	*Hookworms*	*Strongyloides* spp*.*
Overall prevalence (95% CI)	4.0 (1.6–6.4)	8.4 (5.0–11.7)	13.0 (9.1–17.1)	27.4 (21.4–33.4)
Overall prevalence HEE (95% CI)	7.8^a^ (3.3–12.4)	12.4^a^ (6.9–18.0)	14.4^a^ (8.5–20.3)	21.7^a^ (12.9–30.4)
HEE Male (95% CI)	5.8 (0.3–11.2)	14.9 (6.9–23.0)	17.2 (8.7–25.8)	29.0 (16.1–42.0)
HEE female (95% CI)	10.8 (2.5–19.1)	9.2 (1.4–17.0)	10.8 (2.5–19.0)	11.9 (0.9–22.9)
Overall prevalence LEE (95% CI)	0^b^	4.0^b^ (0.6–7.7))	11.7^a^ (6.1–17.3)	31.6^a^ (23.3–40.0)
LEE male (95% CI)	0	3.8 (0–8.7)	17.7 (8.7–26.8)	27.5 (16.2–38.8)
LEE female (95% CI)	0	4.5 (0–10.2)	4.5 (0–10.2)	35.9 (23.4–48.5)

*HEE* is high elevation ecozone; *LEE* is low elevation ecozone. Different superscript (^a,b^) within row indicates significant differences *P* < 0.05

The overall prevalence of any STH based on the Wisconsin technique was 31.5% (95% CI = 26–37%). Prevalence in the LEE was 29.0% (95% CI = 21–37%) and in the HEE 34.0% (95% CI = 26–42%; *P* = 0.384). Overall STH prevalence in males was 34.3% (95% CI = 27–42%) versus 28.2% (95% CI = 20–36%) in females (*P* = 0.315).

Within the ecozones, there was no significantly difference in prevalence between sexes so the data within an ecozone were merged. The HEE had significantly higher prevalence of *Ascaris* sp. and *Trichuris* sp. (*P* < 0.000 vs. *P* = 0.010 respectively, Table 3), compared to the LEE.

Based on a subset of samples (*N* = 62) that were processed using both the Wisconsin and Kato-Katz techniques, prevalence was significantly lower for the KK (32.2%; 95% CI = 20–44%) compared to the Wisconsin (43.5%; 95% CI = 30.4–56.7%) technique (*P* = 0.000). The sensitivity of the KK technique was 63% and specificity was 91% relative to the Wisconsin technique (Tables 4 and 5).

**Table 4:** Agreement between Wisconsin and Kato-Katz techniques for soil transmitted helminth egg detection combined

		Wisconsin		
		Positive (N)	Negative (N)	Total (N)
Kato-Katz	Positive (N)	17	3	20
	Negative (N)	10	32	42
	Total (N)	27	35	62

**Table 5:** Prevalence (95% confidence interval) comparison of three soil transmitted helminths detected (*N* = 65) with Wisconsin and Kato-Katz in the Ngorongoro Conservation Area

	*Ascaris* sp.	*Trichuris* sp.	Hookworms
Overall prevalence			
Wisconsin (95% CI)	3.2 (0–8.4)	11.3 (2.6–20.0)	19.4 (8.7–30.0)
Prevalence			
Kato Katz (95% CI)	3.1 (0–8.0)	7.7 (0.4–14.9)	3.1 (0–8.0)

### Parasitological results: intensity

Intensity, as expressed by eggs per gram (EPG) and larvae per gram (LPG), is summarized in Table 6. Additional observations were low intensities for *Enterobius* spp. in all sex and ecozone groups (*n* = 8), and one female in HEE with 1.0 EPG for unspecified trematode eggs. The univariate linear regression identified no significant correlation between the four STH intensities and either BMI classification or age (y). *Ascaris* sp. was only observed in the HEE with a significantly higher intensity in male vs female (*P* = 0.03). However, for *Trichuris*, a significant difference was observed between males in both ecozones (*P* = 0.03) and females in both ecozones (*P* = 0.01). Overall, the intensity of *Trichuris* sp. and hookworms was significantly different in HEE vs. LEE when combined for both sexes (*P* = 0.001 vs. *P* = 0.05 respectively; Table 6).

**Table 6:** Intensity (median EPG/LPG; min-max) of four soil transmitted helminths with respect to elevation and sex of children in the Ngorongoro Conservation Area

	*Ascaris* sp.	*Trichuris* sp.	Hookworms	*Strongyloides* spp.
Overall intensity (Min-Max)	165.3 (0.6–8653.9)	5.1 (0.2–61.8)	0.6 (0.2–10.2)	0.2 (0.2–4.7)
Infected children	*N* = 12	*N* = 19	*N* = 22	*N* = 21
Intensity HEE (Min-Max)	165.3 (0.6–8653.9)	6.4 (0.4–61.8)	0.4 (0.2–9.8)	0.2 (0.2–2.2)
HEE^*^ male (Min-Max)	242.7 (188.9–8653.9)	5.5 (0.4–61.8)	0.4 (0.2–9.8)	0.4 (0.2–2.2)
HEE female (Min-Max)	80.8 (0.6–231.4)	7.0 (2.3–34.3)	0.4 (0.2–3.9)	0.2 (0.2–0.2)
Infected children	*N* = 0	*N* = 6	*N* = 17	*N* = 42
Intensity LEE (Min-Max)		0.7 (0.2–5.1)	0.8 (0.3–10.2)	0.2 (0.2–4.7)
LEE^o^ male (Min-Max)	0	0.4 (0.2–5.1)	1.0 (0.4–8.2)	0.2 (0.2–4.7)
LEE female (Min-Max)	0	0.8 (0.6–1.7)	0.8 (0.4–10.2)	0.2 (0.2–2.2)

*HEE* is high elevation ecozone; *LEE* is low elevation ecozone; *EPG* Eggs per gram; *LPG* Larvae per gram

Finally, co-infections were compared between the sexes in the two ecozones, and no significant difference were found (Table 7).

**Table 7:** Co-infection with soil-transmitted helminths (STH) of children in the Ngorongoro Conservation Area with respect to elevation and sex

	LEE male	LEE female	HEE^*^ male	HEE female	Total
No STH	53	50	56	44	203
One STH	23	14	23	16	76
Two STH	3	2	7	5	17
Three STH	0	0	1	0	1

*HEE* is high elevation ecozone; *LEE* is low elevation ecozone

### School survey

Water accessibility was evaluated by asking each headmaster to identify the main source of water for their school during rainy and dry seasons. In the LEE, all three schools collected rainwater during the rainy season; two schools also sent children to retrieve water from the river, whereas one pumped water from the river (when diesel was available to operate the pump). None of the schools tested water quality or treated water. In the HEE, two of the three schools got their water from the river and the other got it from water sources within the Ngorongoro crater. For all three HEE schools, water was transported by pipes, but only the school that got water from the crater treated the water with chlorine.

At all six schools, latrine facilities were available with cleanable cement floors and roofing. Although latrine-cleaning routines were practiced, in three out of six facilities fecal contamination of the environment was observed. Hand-washing facilities were only available close to the latrine in one school in LEE, using a small plastic bucket filled with water. Another school had a washing facility that was broken at the time of the study visit. Soap was only available in one school. In the HEE, one school had a tippy tap (large plastic containers hanging from a tree) and the other had a water bucket.

An antiparasitic program (albendazole) was rolled out in 2012 (2 years prior to the study) at two LEE schools and at one HEE school. For the three other schools, data on deworming were not available. Sanitation and hygiene education were confirmed to be included in science classes at LEE schools.

### Checklist results

Two schools had “improved traditional pit latrine” and four had a “VIP latrine,” where VIP latrines are defined as cement floor supported by reinforced iron bar or wood, wood and mud plastered or brick or block wall, corrugated iron roof or thatch roof cover, ventilated through installed vent pipes (Appendix 2). No cleaning material was visibly available. Fecal material was noticed outside the pit, on the floor, or on the walls at four schools. Females’ facilities appeared to be cleaner than those for males. Cleanliness level varied among schools; with some in need of sanitizing, whereas others appeared to have been recently cleaned.

## Discussion

This cross-sectional study generated estimates on prevalence and intensity of four STHs among primary school children in six schools in the NCA. School-based anti-parasitic programs had not been implemented within at least 6 months in any of these schools, ensuring that recent treatments would not affect the results. STH prevalence estimations for a subset of samples analyzed using the Kato-Katz, the WHO recommended diagnostic test, was 32.3%. Based on this, the study area in the NCA would be classified as a medium risk area, where periodic treatment recommendations should be based on diagnostic testing in the different ecozones. When prevalence estimations are > 50%, WHO recommends a bi-annual treatment protocol [9]. With the more sensitive technique based on the Wisconsin protocol, our prevalence estimation in the same subset was 43.5%, and the overall prevalence estimation based on all available samples was 31.5%, neither exceeding the prevalence cut-off for high-treatment frequencies. Kato-Katz is the most frequently used technique in the field; however, other studies have identified the challenges with limited sensitivities of KK [35, 39] and have compared KK to FLOTAC or PCR technologies. Knopp et al. [35] reported the FLOTAC being more sensitive to detect hookworms and *S*. *stercoralis* compared to KK. They also found KK to be equally sensitive as PCR for these same parasites, whereas Verweij et al. [39], reported a higher sensitivity using PCR. Kato-Katz is not recommended for *Strongyloides* spp. and various versions of the Baermann technique have been used to improve its sensitivity [35]. In our study, using an adjusted Baermann technique, the overall prevalence of *Strongyloides* spp. was 27.4%, higher than reported in studies in Angola (11.3%) and on Zanzibar (10.8%) [40, 41]; both studies of which also used a version of the Baermann technique. While these differences may be in part be linked to differences in the climate, environment, and lifestyle (pastoralist in the current study), the different methods for fecal analysis likely play an important role as well. Both Knopp and de Alegria’s studies used a modified Baermann funnel technique which is known to be less sensitive than the beaker-Baermann technique used in this study [29]. Additionally, in Knopp’s study, the Baermann funnels were set up for only 2 h compared to at least 8 h in the current study; restricting the time available for larvae to migrate out of feces and end up in the sediment.

The high prevalence of *Strongyloides* spp*.* may be attributed to the plasticity in its lifecycle that may allow it to persist under adverse conditions. The LEE had the highest prevalence estimations of *Strongyloides* spp., similar to small-scale studies in rural Côte d'Ivoire also at lower elevation (e.g., Lake Taboo at 400 m) [42], emphasizing the potential role of the local climatological differences.

Amor et al. also emphasized the Baermann or combined diagnostics for *Strongloides* detection, and also stated that albendazole, which is the common anthelminthic used in school programs in Tanzania, is not the treatment of choice for *Strongyloides* spp. [43]. This may also contribute to the higher prevalence of this STH observed in this study, as albendazole is the anti-parasitic drug of choice in the school programs [12]. Finally, the WHO does not include Strongyloides spp. infections in the neglected tropical diseases, and might therefore not get the attention this STH deserves in prevention and control (ref 1; new WHO included).

Despite the use of the more sensitive Wisconsin technique, we detected a low prevalence of *A*. *lumbricoides* of 4.0%, and only detected it in the HEE. These results are comparable to data from Angola in children (5–14 years) where also a low prevalence of 1.3% was reported [41], but lower than those in southwest Ethiopia where prevalence was 39.5% [44] and in northwest Ethiopia where prevalence was 39.8% [45]. The reasons for the low prevalence in our study, and the absence from the LEE are unknown, but may reflect a successful mass treatment program. Although our data did not have sufficient spread in age categories, age susceptibilities have been reported in the past [46, 47].

Our main interest in comparing ecozones was motivated from the expected survival of STH eggs and larvae in the more favorable climate of the cooler and more humid HEE. Climate and seasonality can have considerable impacts on transmission dynamics, with significant interannual and within region fluctuations occurring depending on precipitation and temperature [48, 49]. Understanding these ecological drivers and dynamic patterns is needed in order to guide intervention strategies. Summarizing, indeed the overall prevalence in the HEE was higher with 34.0%, and significantly higher prevalence of *Ascaris* sp. and *Trichuris* sp. compared to LEE.

Finally, when reflecting on the potential difference of STH infections in sex, behaviors based on roles in the community should be taken into account. Males had a significantly higher prevalence of *Trichuris* sp. compared to females in the present study. In other settings, males were also identified at higher risk of infection compared to females, and this may be driven by different behaviors associated with their different roles in society (herding vs doing chores near the home) [50–52].

In our study, we observed evidence of possible low hygiene and sanitation in the schools. For instance, hand washing facilities were present in three schools only, and no soap or ash was available for hand washing at any of the six schools. Another study in the same area in the NCA, including both ecozones, identified low hygiene standards in the same communities [18]; however, the NCA would all classify as rural setting and therefore not reflecting hygiene standards more common in urban settings. Substandard hygiene associated with the latrine facilities in schools may lead to fecal contamination of hands with helminth eggs, facilitating the spread of parasitic infections. The survey of headmasters and school inspections revealed that, despite implementation of health and sanitation in the curriculum, many schools struggled to keep infrastructure and basic hygiene facilities functional and available to children as well as present these components in of the curriculum to the students. Support for schools that increases hygiene status is likely to reduce the probability of STH infections in children. In addition, other local and cultural factors, such as eating with fingers as opposed to utensils, and/or behavioral factors, such as walking barefoot and hand hygiene, were not considered in this study and can impact transmission.

As with many field studies, this study has limitations. Only six of 20 public schools were enrolled, and no private schools were included. Only children attending schools participated in the study resulting in a biased sample that may not be representative of all children in this age range in this part of the NCA. Childrens’ ages were self-reported by teachers who based their reporting on incomplete school records. Also, we limited the age range to 8–12, thus results will not be representative of younger or older children. Finally, the study might lack power to find potentially existing differences, as some sex and age groups have lower numbers of participants or observed prevalence and intensities were low. Additionally, the study occurred at a single point in time (following the “short” rains) and did not span different seasons. Given the seasonality of most parasites, our study may have over, or underestimated, depending on the parasite species, the annual parasite burden in this region.

We did not inquire about clinical signs consistent with parasitism that children may have experienced. This additional information could inform the knowledge of STH infection symptoms as well as the interference with other common diseases within the list of neglected tropical diseases. Further surveys could include questions on education of parents and older siblings, health, and sanitation routines outside of school (e.g., clipping of fingernails, the presence of livestock on the home property, handwashing, latrine facilities, and wearing shoes).

## Conclusions

In conclusion, significant differences in prevalence in high and low ecozones exist, and STH infections are still very common among school children suggesting that anthelminthic intervention and education may be necessary in this region.

## Data Availability

The datasets generated and/or analyzed during the current study are not publicly available due to privacy reasons but are available on basis of anonymity from the corresponding author on reasonable request. There are no materials available.

## Appendix 1

Primary School Interview Form

Interviewer ID:

Date of Interview:

Total number of child forms collected From ID# to ID#

1. General Information

Name of District: Name of School:

Name of School Headmaster:

Name of Deputy School Director: Telephone Number of Headmaster:

Distance from town/village km

Distance to closest dispensary/hospital km GPS coordinates

2. Student and Teacher Population

**Table:**

	Total	No. of Males	No. of Females
Students			
Teachers			
Performance on St. 7 exam			
% transitioning to secondary school			

3. Classrooms

Total number of classrooms ________________

Average number of pupils per classroom

4. Water Supply

- Please describe the water supply at the school. What is the source? How far away does it come from? How is it stored?
- Is the water tested, or treated before use? If yes, how?

5. Hand Washing Facilities

- Does the school have hand washing facilities? If so, what kind? (Sink and faucet, bucket, basin, tippy tap)
- Is their soap/ash available to wash hands?
- When do students wash hands?

6. Latrine Facilities

- Are there latrines at the school? If no, where do students and teachers go to urinate/defecate?
- Is there a latrine-cleaning program? If yes, who cleans the latrines? How often?

7. School Feeding Program

- Please describe the current school feeding program (who pays for it, what food is provided, which meals)
- Please describe the school feeding program in the past (who paid for it, what food was provided, which meals, if it was ever interrupted)
- Please describe any future plans or goals for the school feeding program.
- Does the school feeding program need improvement? If so, how could it improve?

8. School Deworming Program

- Is there a deworming program in place at this school? If yes, who pays for it and who administers the drugs to students?
- What drugs are given to children? How often? When were drugs last administered at this school?
- Do you know if children that are not in school get deworming medications? If so, how?
- Do you feel the deworming program is effective? If not, how could it be improved?

9. School Hygiene Lessons
  - Is hygiene education part of the school curriculum? If yes, which standards receive lessons on hygiene? Is hand washing taught in the lessons?
  - Are staff trained in providing hygiene education?
  - Are there supporting teaching materials available?
  - Are there any extracurricular activities or school clubs that include hygiene activities?
  - Are there any posters, other IEC materials with hygiene messages on walls?

## Appendix 2

Primary School Observation Checklist

1. Latrine Facilities

**Table:**

1	What types of latrines are the school (mark one): o None o Traditional pit latrine (dirt floor supported by logs of wood, wood walls with mud plastered, corrugated iron roof or thatch o Improved traditional pit latrine (washable cement floor, supported by logs or reinforced, wood and mud walls, corrugated iron or thatch roof) o VIP latrine (cement floor supported by a reinforced iron bar or wood, wood and mud plastered or brick or block wall, corrugated iron roof or thatch roof cover, ventilated through installed vent pipes)		
3	Are there separate facilities for boys and girls?	Yes	No
4	Number of squat holes available for boys		
5	Number of squat holes available for girls:		
6	Do boys have urinals?	Yes	No
7	Do facilities have doors or curtains for privacy for boys? Do facilities have doors or curtains for privacy for girls?	Yes Yes	No No
8	Can facilities be locked for safety and privacy?	Yes	No
9	Is there wiping material available in the facilities?	Yes	No

2. Latrine Cleanliness

**Table:**

1	Is there fecal material deposited outside the pit or on the floor?	Yes No
2	Anal cleaning material or toilet paper on floor?	Yes No
3	Additional comments/observations	

3. Hand Washing Facilities

**Table:**

1	Are hand washing facilities near the latrines?	Yes No
2	Is there water in the containers?	Yes No
3	Is there soap, ash, or other near the wash stand?	Yes No
4	Is there any reminder for hand washing near latrine?	Yes No

5. School compound

Comments/observation

## Abbreviations

BMI: Body mass index
CHREB: Conjoint health research ethics board
CI: Confidence interval
COSTEC: Tanzania commission for science and technology
CUHAS: Catholic university of health and allied sciences
DEO: District education officer
DMO: District medical officer
EPG: Eggs per gram
HEE: High elevation ecozone
KK: Kato-katz
LEE: Low elevation ecozone
LMIC: Low- and middle-income countries
LPG: Larvae per gram
Max: Maximum
Min: Minimum
NCA: Ngorongoro conservation area
NIMR: National institute of medical research
spp.: Species
SPSS: Statistical package for social science
STH: Soil transmitted helminths
WHO: World health Organization
